# Isolation and Characterization of Avirulent and Virulent Strains of *Agrobacterium tumefaciens* from Rose Crown Gall in Selected Regions of South Korea

**DOI:** 10.3390/plants8110452

**Published:** 2019-10-25

**Authors:** Murugesan Chandrasekaran, Jong Moon Lee, Bee-Moon Ye, So Mang Jung, Jinwoo Kim, Jin-Won Kim, Se Chul Chun

**Affiliations:** 1 Department of Food Science and Biotechnology, Sejong University, Gwangjin-gu, Seoul 05006, Korea; chandrubdubio@gmail.com; 2 Department of Environmental Health Science, Konkuk University, Gwangjin-gu, Seoul-143 701, Korea; moon032@korea.ac.kr (J.M.L.); ruirui26@konkuk.ac.kr (B.-M.Y.); hopesm929@naver.com (S.M.J.); 3 Institute of Agriculture & Life Science and Division of Applied Life Science, Gyeongsang National University, Jinju 52828, Korea; jinwoo@gnu.ac.kr; 4 Department of Environmental Horticulture, University of Seoul, Seoul 02504, Korea; jwkim@uos.ac.kr

**Keywords:** *Agrobacterium tumefaciens*, crown gall, opines, rose, pathogenicity

## Abstract

*Agrobacterium tumefaciens* is a plant pathogen that causes crown gall disease in various hosts across kingdoms. In the present study, five regions (Wonju, Jincheon, Taean, Suncheon, and Kimhae) of South Korea were chosen to isolate *A. tumefaciens* strains on roses and assess their opine metabolism (agrocinopine, nopaline, and octopine) genes based on PCR amplification. These isolated strains were confirmed as *Agrobacterium* using morphological, biochemical, and 16S rDNA analyses; and pathogenicity tests, including the growth characteristics of the white colony appearance on ammonium sulfate glucose minimal media, enzyme activities, 16S rDNA sequence alignment, and pathogenicity on tomato (*Solanum* *lycopersicum*). Carbon utilization, biofilm formation, tumorigenicity, and motility assays were performed to demarcate opine metabolism genes. Of 87 isolates, 18 pathogenic isolates were affirmative for having opine plasmid genes. Most of these isolates showed the presence of an agrocinopine type of carbon utilization. Two isolates showed nopaline types. However, none of these isolates showed octopine metabolic genes. The objectives of the present study were to isolate and confirm virulent strains from rose crown galls grown in the different regions of Korea and characterize their physiology and opine types. This is the first report to describe the absence of the octopine type inciting the crown gall disease of rose in South Korea.

## 1. Introduction

Crown gall disease is considered a ubiquitous plant tumor disease infested by the soil bacterium, *Agrobacterium tumefaciens* [1,2]. The host range of crown gall disease is limited to dicots with few exceptions amongst monocots and gymnosperms. *Agrobacterium* mediates transformation through tumor-inducing plasmid (pTi) harboring *vir* regulon across plant kingdoms efficiently in the realm of molecular plant biotechnology [3] and microbial factories for food production [4]. *Agrobacterium* strains can be classified according to opine utilization into nopaline, octopine, succinamopine, agropine–succinamopine, and chrysopine types. Opine metabolism acts in the co-transfer of oncogenes for efficient tumorigenicity [5,6]. Opine synthesis genes have been illustrated in the T-DNA of pTi/root-inducing plasmids (pRi). They have been proven to encode opine synthesis genes utilizing sugars, ketoacids, and amino acids as substrates [7,8,9,10]. Deoxy fructosyl glutamate (DFG) acts as a carbon and nitrogen source for pTi plasmid-mediated agropine, mannopine, and octopine catabolism, rendering simultaneous activities upon colonization by *A. tumefaciens* [11]. 

Oncogenes in the T-DNA indirectly influence auxin and cytokinin biosyntheses and escalate plant sensitivity to hormone uptake [12]. The upregulation of auxin and cytokinin levels can lead to uncontrolled cell division and tumorigenicity [13]. Tumorigenic abilities and the conjugative transfer of Ti plasmids in biofilm communities of *Agrobacterium* are attributed to both quorum sensing and quenching in opine catabolism [14]. Comparative genomic analyses for the evolutionary relationship among *Rhizobium rhizogenes* plasmids belonging to Ti and opine catabolism plasmids have been performed [15] to elucidate the complex reactions among various opines. These studies indicate that significant variation is present amongst opine metabolism gene subtypes, expressing a dire need for genome-wide analysis for every opine. Octopine-type Ti plasmids include two DNA fragments, namely TL-DNA and TR-DNA, which encode for 13 prominent proteins exhibiting two classes of enzymatic reactions for plant growth promotion and tumorigenicity, in which pyruvate condensation is linked to variations in octopine and nopaline T-DNAs [16]. Octopine uptake and catabolism involve complex transcription regulation and the utilization of LysR-type transcriptional activators OccRs (octopine catabolism regulator) encoded by *occ* genes to alter binding affinities in the bacterium, whereas LacI-type repressors can limit the synthesis of opines such as agrocinopines and mannopine synthesized from sugars [17,18,19]. These complex regulatory networks in opine metabolism pose questions regarding the antagonistic effects within opine components and a competitive inhibition for the synthesis of a particular opine. Insights into octopine-type Ti plasmid pTiA6 have shown genes encoding functions such as plasmid maintenance, virulence, opine catabolism, and conjugative transfer for phytohormone biosynthesis, especially auxins and cytokinins [20].

Octopine expression relies on feedback regulation based on acyl homoserine lactones (AHLs) involving transcriptional activator (TraR), transcriptional anti-activator (TraM) for quorum sensing, and AHL lactonase (AiiB) for quorum-quenching of opine T-DNA responsible for the interkingdom transmissibility profiles of *A. tumefaciens* [21,22,23,24]. TraR and AHL upon an increase in concentration can determine the synthesis of a high copy number of Ti plasmids for disseminating the infectivity and cell proliferation, ultimately leading to active opine catabolism and tumor formation. The negative regulation of AHL is responsible for a decrease in biofilm formation abilities. It is mediated by TlrR and TraM, which in turn suppress TraR activity and BiiA levels [25].

The aim of the present study was to isolate *A. tumefaciens* and assess Ti-plasmid (octopine, nopaline, and agrocinopine) diversities from five different regions (Wonju, Jincheonn, Taean, Suncheon, and Kimhae) in South Korea. There are scarce publications pertaining to the specificity of opine presence in *A. tumefaciens* from South Korea. The further demarcation of phenotypic characterization was performed to determine factors related to opine metabolism in viz., including the utilization of carbon source, and variations in enzymatic activities, pathogenicity, and biofilm formation. Variations in the presence of diversified octopine, nopaline, and agrocinopine reported from various countries were also considered for summarization of the presence or absence of opines.

## 2. Materials and Methods

### 2.1. Plant Samples 

Rose crown gall tissues were collected from five different regions of South Korea: Wonju (37° 19’52.35”, 127° 53’ 30.30”), Jincheon (36° 51’12.46”, 127° 25’ 50.46”), Taean (36° 43’39.18”, 126° 18’ 15.29”), Suncheon (34° 55’22.82”, 127° 29’ 25.63”), and Kimhae (35° 13’ 22.66”, 128° 52’ 57.72”). Samples were immediately transferred to the laboratory. The galls of samples were placed in a refrigerator to reduce contamination until gall extraction. The roses growing in greenhouses were observed to see the galls formed in the stem near the ground of the growth media, which was hydroponic culture with rock wools. The galls were cut out from the stems with sharp knives dipped into 70% ethanol.

### 2.2. Gall Extraction 

Samples were rinsed with tap water to remove soil and hazardous materials. Galls were surface sterilized with 2% NaOCl for 30 min by soaking the galls. After washing three times with sterilized distilled water, galls were finely chopped, immersed in distilled water, and incubated overnight at room temperature (28°C).

### 2.3. Isolation and Phenotypic Characterization of Agrobacterium Isolates

The screening of *Agrobacterium* spp. from rose crown galls in five different regions of South Korea was carried out by using ATGN (glucose minimal medium) media [26]. Bacteria were maintained on *Agrobacterium tumefaciens* (AT) minimal medium supplemented with 0.5% (w/v) glucose and 15 mM of ammonium sulfate (ATGN). The accumulation of iron oxide precipitates was disregarded for FeSO_4_.7H_2_O to minimize adverse growth complications in the initial AT recipe. Overnight incubated crown gall extracts were streaked on ATGN media. Plates were incubated at 28°C for 48 h and examined for growth and color development. Bacterial colonies were selected based on colonies form, elevation, surface, color, and so on. Individual colonies were cultured on ATGN medium and preserved in glycerol (25%) stock for further experimentation. The bacterial colonies were cultured on ATGN or sometimes on Luria-Bertani (LB) media for preservation.

### 2.4. Analysis of 16S rDNA for Bacteria Isolates 

#### 2.4.1. PCR Amplification of 16S rDNA

Representative colonies were analyzed to determine their genera based on 16S rDNA PCR amplification for bacteria. Single colonies were transferred from plates and cultured in 96-well plates with 300 μl Reasoner's 2A (R2A) broth (Difco, USA) at 28°C for approximately 2–3 days with shaking (250 rpm). To extract genomic DNA, colonies cultured in R2A broth were lysed by five times of shock-freezing in liquid nitrogen and thawing at 65°C followed by centrifugation at 4°C (21,055 g, A1.5M-24 Rotor ø172 x 38.5 mm, Hanil Science, Taejeon, S. Korea). Extracted genomic DNA was used for PCR amplification of 16S rRNA gene using universal primers of fD1 (5′-AGAGTTTGATCCTGGCTCAG-3′) as a forward primer and rP2 (5′-ACGGCTACCTTGTTACGACTT-3′) as a reverse primer [27] to determine their genera. The 16S rDNA gene was amplified by PCR in a reaction mixture containing 5 pmol each of primer fD1 and rP2, 0.25 mM dNTP each, and Taq polymerase buffer. The final volume of the PCR mixture was adjusted to 20 μl by adding dH_2_O. Then, 0.25 units of Taq polymerase (Solgent Co., Ltd., Korea) was added to the reaction mixture. Thermal cycling was performed with a T-gradient thermo-block (Biometra^@^GmbH). PCR conditions consisted of one cycle of 95°C for 4 min, 34 cycles of 95°C for 1 min, 58°C for 1 min, and 72°C for 1 min, and finally one cycle of 72°C for 8 min. PCR products were analyzed by electrophoresis on 1% agarose gels and stained with ethidium bromide to detect PCR-amplified DNA fragments.

#### 2.4.2. Sequence Analysis of 16S rDNA

PCR products were purified using a Montage PCR 96 Cleanup kit (Montage, USA) according to the manufacturer's instructions [28]. Then, 16S rDNA sequences were determined with an Applied Biosystems 3100 sequencer (Applied Biosystems) using a 783R primer (5-GTGGACTACCAGGTATCTA-3'). DNA sequence analysis was performed using Ribosomal database project (RDP) [29] and DNASTAR software program [30]. Sequences were aligned together with those of representative members of elected genera by using the CLUSTAL W program [31]. A phylogenetic tree for datasets was inferred with a neighbor-joining method [32] using MEGA version 7.0 [33]. The bootstrap analysis involved 1000 replications of neighbor-joining data to assess stable relationships. 

### 2.5. Enzyme Assay (API ZYM strip) for Utilization Test of Carbon Sources 

The API ZYM (Analytical profile index of enzyme) is a semi-quantitative method used for the rapid study of 19 enzymatic activities. Enzymes were assayed in API-Zymstrip (Bio-Merieux, Marcy-l’Etoile, FR) according to the manufacturer's instructions.

### 2.6. Motility Assay

Swimming and chemotaxis phenotypes were tested on ATGN swim agar plates containing 0.5% agar [34]. Petri plates were filled with 25 mL of ATGN swim agar. Swim plates were inoculated from fresh colonies or cultures using a toothpick that was stabbed into the agar at the center of the plate and incubated at 28°C for 4 days.

### 2.7. Bioassay for Testing Pathogenicity

Abilities of Korean isolates of *Agrobacterium* to infect plants and produce galls were tested on tomato (*Solanum lycopersicum*) plants under greenhouse conditions using standard methods [35]. In this test, the stems (crown) of four tomato seedlings (2 weeks old) for each isolate were wounded with a sharp knife near the soil and inoculated with bacterial culture (O.D. = 1.0 at 600 nm). After 4 weeks, inoculated plants were checked for formations of young galls (tumors) developing from the meristematic tissue around the central vascular system. *A. tumefaciens* C58 cultures were used as positive controls.

### 2.8. Biofilm Assay

Overnight bacterial cultures in ATGN liquid media were diluted with fresh medium to obtain an OD600 of 0.04. Then, 200 μL of diluted culture was placed into 96-well polystyrene or polypropylene microtiter plates and incubated at 28°C. After 48 h of incubation, an aliquot of the liquid culture was initially transferred to a new plate, and then OD600 was measured.

### 2.9. PCR for Genes Related to Carbon Source Utilization

PCR primer sets were used to detect genes for agrocinopine, nopaline, and octopine to characterize isolates regarding the utilization of carbon source. The sequences of primers used for PCR analysis are listed in Table 1.

DNA isolation was carried out according to the mini-prep plasmid isolation kit (Promega, Madison, WI, USA). The genes were amplified by PCR in a reaction mixture containing 10 pmol each of specific primer sets (Table 1), 0.25 mM dNTP (deoxyribonucleotide triphosphate) each, and 2 μL of 10 × Taq polymerase buffer. The final volume of the PCR mixture was adjusted to 20 μL by adding dH_2_O. Then, 0.25 units of Taq polymerase (Solgent Co., Ltd., Korea) was added to the reaction mixture. Thermal cycling was performed with a T-gradient thermo-block (Biometra^@^GmbH). PCR cycles were as follows. (1) Agrocinopine PCR conditions consisted of one cycle of 94°C for 4 min, 34 cycles of 94°C for 1 min, 58°C for 1 min, and 72°C for 1 min, and finally one cycle of 72°C for 5 min. (2) RBF-RBR (nopaline right border forward and reverse) primers; 2 min denaturation at 94°C, followed by 35 cycles at 94°C, 60°C, and 72°C for 1 min at each temperature [36]. (3) ocsF-ocsR (octopine genes forward and reverse) primers; 3 min denaturation at 94°C, followed by 35 cycles of 94°C, 58°C, and 72°C for 1 min at each temperature. The final elongation was at 72 for 5 min [36]. PCR products were analyzed by electrophoresis on 1% agarose gels and stained with ethidium bromide to detect PCR-amplified DNA fragments.

## 3. Results and Discussion

### 3.1. Isolation and Phenotypic Characterization

**Figure 1:**
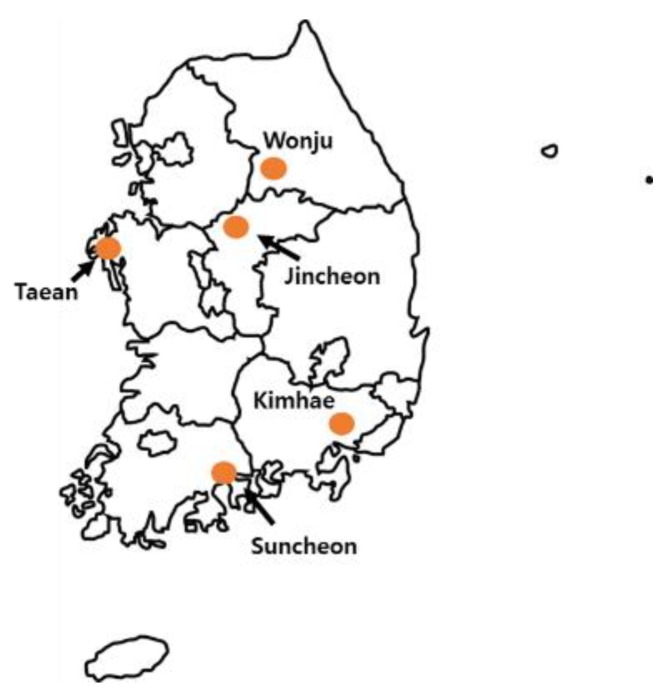
Sampling map in Korea.

The primary objective of the present study was to isolate *A. tumefaciens* strains from different crown galls of rose in five regions of South Korea, namely Wonju, Jincheonn, Taean, Suncheon, and Kimhae (Figure 1). 

Identification and characterization were accomplished based on various morphological, physiological, biochemical, and phytopathogenic tests as well as molecular analysis. Based on their morphological characteristics on ATGN, a total of 87 strains were isolated, and potential virulent strains were identified confirming opine synthesis genes by amplification of the agrocinopine gene fragment and pathogenicity test in the greenhouse. 

Similar works corresponding to the isolation and characterization of *A. tumefaciens* strains for arresting crown gall diseases in rose have been globally carried out [37,38,39]. To understand their biology of the strains, their physiological and biochemical characteristics were assessed based on the variability patterns of 18 enzymatic activities, which might be different between virulent and avirulent strains. Of all isolates, five were alienated for enzyme activities as avirulent or virulent isolates. Then, the biochemical features of these five isolates (RC053, RC084, RC111 for avirulent, RC081, and RC178 for virulent) were determined (Table 2).

Utilization of the amino acid, lipid, and carbon sources was not related to virulence. There was no specific pattern between the avirulent and virulent strains in relation to carbon or nitrogen sources. Most of the *Agrobacterium* strains tested in this study produce esterase, esterase lipase, acid phosphatase, and naphthol-AS-BI-phosphohydrolase. However, leucine, valine, cysteine arylamidase, alpha and beta-galactosidase, alpha and beta-glucosidase, N-acetylglucosaminidase, alpha-mannosidase, alpha-fucosidase activities were different depending on the strains. All these enzymes were known to be produced from most of bacteria (Swiss UniProtKB database).

The pathogenicity of virulent *Agrobacterium tumerfaciens* is well known to be dependent on the pTi plasmid, which has vir genes, and also the gene for synthesizing and utilizing opines as carbon sources [13,19,40,41]. However, the biochemical characteristics for other carbon or nitrogen sources between virulent and avirulent strains have not been studied much previously.

### 3.2. Biofilm Formation

**Figure 2:**
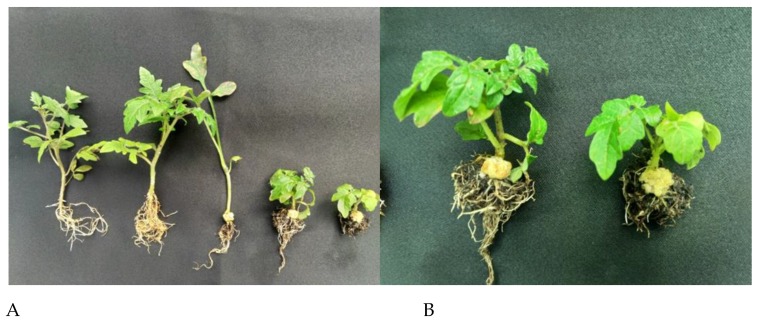
The tumor-forming ability of selected isolates on tomato in the greenhouse. (**A**). Healthy to tumor formed from the left of pathogenic isolates. (**B**). Close-up of tumor formation with *Agrobacterium tumefaciens* C58.

The biofilm-forming abilities of selected isolates are listed in Table 3. From these results, it was evident that RC008, RC009, RC011, RC012, RC013, RC014, RC026, RC027, RC029, RC030, RC032, RC081, RC132, RC133, RC134, RC135, RC140, RC170, and RC178 were pathogenic strains by the agrocinopine gene amplification of pTi and gall formation in the greenhouse (Figure 2 and Table 3).

RC008–RC012 all showed crown gall formation in the tomato plants, and the agrocinopine gene was amplified [42]. However, the biofilm-forming abilities did not contribute to virulence. Even the strains with low biofilm-forming abilities still maintained the virulence in tomato plants. Whereas RC066, RC112, RC141, RC067, and RC068—which had good biofilm-forming abilities based on their absorbance values—were avirulent (Table 3). Biofilm formation and the pathogenicity profiles of various *Agrobacterium* strains have revealed quorum-sensing mechanisms as the primary phenomenon for upregulating the copy number of Ti plasmids to maximize pathogenicity, which consequently hacks phytohormones such as salicylic acid, indole acetic acid, and ethylene, resulting in the downregulation of *vir* gene expression [43]. Opine-type Ti plasmids such as nopaline/agrocinopine and octopine/mannityl opines comprise *try* and *tra* operons for effective gene regulation to control conjugative transfer and quorum sensing, under which circumstances operons regulating agrocinopine activities can also result in octopine conjugation controls [44]. Furthermore, quorum sensing regulatory cascades upon environmental interactions can also restore the virulence of non-pathogenic strains [45,46]. Similarly, when the conjugal transfer of opines in the catabolic plasmid pAtK84b was assessed for nopaline and agrocinopines A and B, it was found that *traR* was present in duplicate copies, contributing to genetic transfer without the induction of either of the two opines used, in which one copy deleted nopaline metabolism, while another copy was induced by agrocinopines A and B without the involvement of nopaline [47]. 

Among various isolates, RC088 and RC098 revealed escalated motility patterns on the fourth day to a diameter of 9.0 cm compared to the initial movement with a diameter of 4.0 cm. Comparatively, RC036 and RC116 also depicted increased motility with a diameter of 8.5 cm from a diameter of 3.5 cm observed on the first day. RC016, RC017, and RC049 failed to show significant variations in motility patterns. Nevertheless, there were prominent variations among all the isolates in motility patterns, stressing the role of motility profiling for emphasizing the pathogenicity and tumorigenicity properties of these bacteria. Therefore, the ability to form motility in *A. tumefaciens* was strain-specific. Opine catabolism in *A. tumefaciens* is known to be correlated to chemotaxis [44]. Hence, further in-depth analyses of chemotaxis and motility are needed to confirm the chemotactic profiles of individual isolates for corroborating opine synthesis and catabolism. 

### 3.3. Tumor Formation

Initial infection with *A. tumefaciens* transforms host plants for increased cell proliferation and tumor formation regulated by opine metabolism for energy requirements. Crown galls can act as a sink to provide specific nutrient requirements for efficient tumor formation backed by metabolic product accumulation in these galls [48]. The tumorigenicity of *A. tumefaciens* was confirmed based on biochemical characteristics and tumor-forming abilities, as illustrated in Figure 2. In South Korea, disease severity is approximately 10%–20%, depending on the region and greenhouse.

Ti plasmid mutants initially were characterized by tumor formation and octopine catabolism [49]. Later, biocontrol strategies for combating crown gall disease when comparatively employing *A. radiobacter* K84 and the *Tra* mutant strain K1026 significantly proved that K1026 was a safe biocontrol agent. The remarkable presence of both nopaline and octopine metabolism genes in both strains utilized for studies provided inquisitive roles for opine metabolism and biocontrol efficacies [50]. Recently, niche construction has been found to be regulated through the distinct ligand-binding patterns of a periplasmic binding protein (PBP) NocT with ligands nopaline and pyronopaline for the entrapment of opines in *Agrobacterium,* confirming the uniqueness in the binding, transport, and nutrient assimilation in this bacterium after tumorigenesis [51]. An excellent review of the historical perspectives of tumorigenesis in crown galls has revealed tumor induction and niche establishment [52]. 

### 3.4. Genes Associated with Opine Metabolism

**Figure 3:**
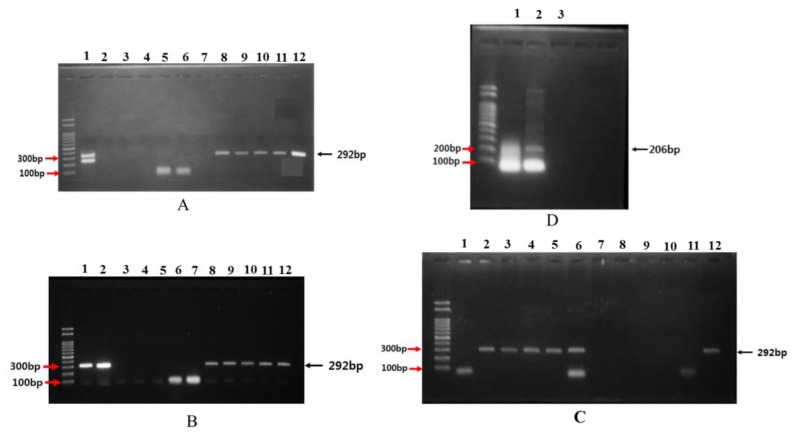
PCR amplification of gene fragments of agrocinopine, nopaline, and octopine of *Agrobacterium tumefaciens* RC strains. (**A**) From left lane: 1 kb size marker, Lanes 1 (C58), 2 (RC002), 3 (RC003), 4 (RC004), 5 (RC005), 6 (RC006), 7 (RC007), 8 (RC008), 9 (RC009), 10 (RC010), 11 (RC011), and 12 (RC012); (**B**) From left lane: 1 kb size marker, Lanes 1 (RC013), 2 (RC014), 3 (RC016), 4 (RC017), 5 (RC018), 6 (RC019), 7 (RC024), 8 (RC026), 9 (RC027), 10 (RC029), 11 (RC30), and 12 (RC032); (**C**) From left lane: 1 kb size marker, Lanes 1 (RC131), 2 (RC132), 3 (RC133), 4 (RC134), 5 (RC135), 6 (RC140), 7 (RC141), 8 (RC146), 9 (RC160), 10 (RC162), 11 (RC165), and 12 (RC170); (**D**) From left lane: 1 kb size marker, Lanes 1 (RC081) and 2 (RC178). Agrocinopine and nopaline gene fragments were amplified, showing sizes of 292 bp and 206 bp, respectively. Gene fragments were sequenced and confirmed through sequence alignment (CLC Workbench 5.0) as the right sizes of genes for sucrose and L-arabinose phosphodiester synthesis in tumor inducing plasmid (pTi).

PCR primers for agrocinopine and nopaline in *A. tumefaciens* RC strains are amplified and depicted in Figure 3. PCR amplification of gene fragments for agrocinopine and nopaline in Ti plasmids were 292 bp and 206 bp, respectively.

**Figure 4:**
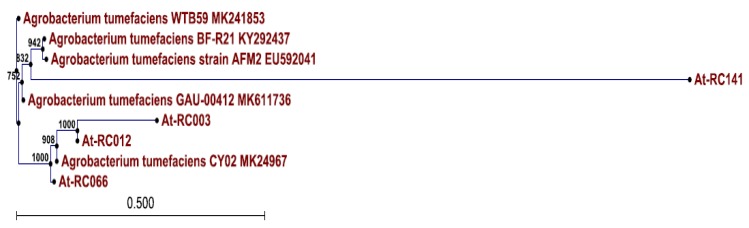
Neighbor-joining tree based on 16S rDNA sequences of isolates. Standard reference strains have strain numbers followed by Genebank accession no. RC012 and RC066 were pathogenic, whereas RC003 and RC141 were nonpathogenic isolates of *A. tumefaciens*. Bootstrap values based on 1000 replications are indicated as numbers in internodes.

The sequences of the DNA fragments were confirmed through alignment in BLAST (Data not presented) as the right sizes of genes for sucrose and L-arabinose phosphodiester synthesis in pTi. The presence of the above-mentioned sugar and amino acid is the incumbent property of agrocinopines A and B involved in phosphodiester linkages. Of 87 positive isolates, 18 isolates (RC008, RC009, RC010, RC011, RC012, RC013, RC014, RC026, RC027, RC029, RC030, RC032, RC132, RC133, RC134, RC135, RC140, and RC170) had agrocinopine types of gene amplification. Two isolates (RC081 and RC178) had nopaline-type gene amplification. None of these isolates had octopine-type gene amplification. This indicates that Korean isolates probably do not carry octopine-type Ti plasmids. Neighbor-joining tree analysis also showed distinct variations among pathogenicity linking to amplified isolates, revealing that RC012 and RC066 were pathogenic, whereas RC003 and RC141 were non-pathogenic isolates of *A. tumefaciens*. However, lineages showed unique variations for RC141 and other non-pathogenic isolates with the possibility of a common ancestor (Figure 4). 

Of 87 isolates, 18 pathogenic isolates showed the agrocinopine type of carbon utilization in Korean rose. Among these isolates, two with the nopaline type were tested. There was no octopine type in this study. Similar results were obtained in a study for the identification of opine types in Japan, showing that some isolates belonged to neither the nopaline nor octopine type after experimental studies [53]. In the pursuit of analysis for the absence of octopine in these isolates, octopine degradation was proposed as the principal phenomenon to address this issue. Further genes encoding octopine degradation were not needed for tumorigenesis in *A. tumefaciens* [54]. The conjugative transfer of Ti plasmids has also been reported to promote opine degradation because of deficient levels of AHLs [55]. The enzymatic degradation of nopaline, octopine, and octopine acid involves the bioactivity of opine permease or opine oxidase that regulates the catalysis of amino acid biosynthetic pathways of arginine and ornithine [56,57]. Furthermore, ample research cites that octopine is not necessary for tumorigenesis in *A. tumefaciens* [58]. Enzymatic degradation assessment of mannopine and agropine transport systems employing *Bam*HI has shown that only the AGR system is more susceptible to transient growth characteristics of *A. tumefaciens* than the mannopine (MOP) transport system [59]. These explanations shed preliminary insights into octopine deletion among various isolates of South Korea. To the best of our knowledge, the characterization of opine gene types in the Ti plasmids of *A. tumefaciens* in crown galls of Rose has not been reported earlier. The present study can be regarded as the first report of octopine genes in South Korea. To further characterize the presence/absence of octopine genes in various regions, a literary survey was conducted.

## 4. Conclusions

The *A. tumefaciens* were successfully isolated from rose plants in the five regions of S. Korea. Most of the strains from the galls of roses were not virulent. There was no difference in the capabilities of biofilm formation, and carbon utilization is related to virulence between avirulent and virulent isolates. Virulence was solely dependent on pTi that has opine synthesis and the utilization gene, which was confirmed by the gene amplification of agrocinopine. Most Korean isolates of *A. tumefaciens* were the opine type with a few nopaline types. However, there was no octopine type from Korean isolates in this study. To our best knowledge, this study was first report on the types of opine present in S. Korea.

## Figures and Tables

**Table 1:** List of primers used in this study.

Gene	Primer Name	Primer Sequence	Reference
Agrocinopine	ACC-F	5’ AGGAATGAAAATGAACCCTCT 3’	In this study
	ACC-R	5’ CTCCGAACTGAACCAACTCCC 3’	
Nopaline	RB-F	5’ TGACAGGATATATTGGCGGGTAA 3’	[36]
	RB-R	5’ TGCTCCTCCGTCAGGCTTTCCGA 3’	
Octopine	OCS-F	5’ ATGGCTAAAGTGGCAATTTTGGG 3’	[36]
	OCS-R	5’ TCAGATTGAASTTCGCCAACTCG 3’	

**Table 2:** Enzymatic activities of avirulent and virulent isolates from crown gall.

Enzyme	*Agrobacterium tumefaciens* Strains ^a^				
	RC ^b^ 053	RC ^b^ 084	RC ^b^ 111	RC ^c^ 081	RC ^c^ 178
Alkaline phosphatase	−	+	+	+	+
Esterase	+	+	+	+	+
Esterase lipase	+	+	+	+	+
Lipase	+	−	+	−	+
Leucine arylamidase	−	−	+	+	+
Valine arylamidase	+	+	−	+	−
Cystine arylamidase	−	+	+	−	+
Trypsin	−	−	+	+	+
a-chymotrypsin	+	−	+	−	−
Acid phosphatase	+	+	+	+	+
Naphthol-AS-BI-phosphohydrolase	+	+	+	+	+
a-galactosidase	−	−	+	−	+
b-galactosidase	+	+	+	+	−
b-glucuronidase	−	−	+	−	+
a-glucosidase	−	−	+	−	+
b-glucosidase	−	+	+	+	+
N-acetyl-b-glucosaminidase	+	−	+	−	+
a-mannosidase	−	−	+	−	+
a-fucosidase	+	−	−	−	−

^a^ The other isolates were not tested for this. We tested to know variability in the enzyme activities of *A. tumerfaciens* isolates using five different isolates; ^b^ Avirulent strains, ^c^ Virulent strain.

**Table 3:** Biofilm formation and pathogenicity of *A. tumefaciens* isolate from rose crown gall.

Strain ^a^	Abs ^b^. (Mean ± std.)	Gall formed ^c^ (+, −)	Strain	Abs. (Mean ± std.)	Path (+, −)	Strain	Abs. (Mean ± std.)	Gall formed (+, −)
C58	0.49 ± 0.13	+	RC076	0.11 ± 0.01	−	RC132	0.21 ± 0.04	+
RC002	0.78 ± 0.15	−	RC077	0.10 ± 0.01	−	RC133	0.12 ± 0.01	+
RC003	0.61 ± 0.23	−	RC079	0.13 ± 0.04	−	RC134	0.18 ± 0.05	+
RC004	0.61 ± 0.18	−	RC080	0.12 ± 0.01	−	RC135	0.27 ± 0.01	+
RC005	0.61 ± 0.04	−	RC081	0.23 ± 0.00	−	RC140	0.18 ± 0.03	+
RC006	0.61 ± 0.13	−	RC082	0.32 ± 0.16	−	RC141	0.85 ± 0.15	−
RC007	0.71 ± 0.11	−	RC083	0.09 ± 0.01	−	RC160	0.61 ± 0.11	−
RC008	0.42 ± 0.03	+	RC084	0.27 ± 0.04	−	RC162	0.14 ± 0.04	−
RC009	0.26 ± 0.04	+	RC085	0.14 ± 0.01	−	RC165	0.12 ± 0.02	−
RC011	0.14 ± 0.02	+	RC087	0.07 ± 0.01	−	RC170	0.09 ± 0.01	+
RC012	0.61 ± 0.18	+	RC088	0.14 ± 0.02	−	RC171	0.11 ± 0.02	−
RC013	0.55 ± 0.11	+	RC089	0.11 ± 0.02	−	RC172	0.08 ± 0.01	−
RC014	0.56 ± 0.07	+	RC090	0.13 ± 0.01	−	RC173	0.10 ± 0.01	−
RC016	0.60 ± 0.08	−	RC091	0.16 ± 0.02	−	RC174	0.09 ± 0.01	−
RC017	0.10 ± 0.01	−	RC092	0.11 ± 0.03	−	RC175	0.10 ± 0.02	−
RC018	0.53 ± 0.30	−	RC093	0.12 ± 0.02	−	RC178	0.48 ± 0.05	+
RC019	0.17 ± 0.01	−	RC096	0.25 ± 0.04	−	RC179	0.11 ± 0.01	−
RC026	0.09 ± 0.00	+	RC096	0.25 ± 0.04	−	RC180	0.15 ± 0.02	−
RC027	0.22 ± 0.03	+	RC098	0.18 ± 0.03	−			
RC029	0.11 ± 0.01	+	RC099	0.08 ± 0.00	−			
RC030	0.08 ± 0.01	+	RC101	0.10 ± 0.01	−			
RC032	0.17 ± 0.02	+	RC103	0.10 ± 0.00	−			
RC033	0.13 ± 0.03	−	RC111	0.20 ± 0.05	−			
RC036	0.16 ± 0.02	−	RC112	0.87 ± 0.61	−			
RC049	0.13 ± 0.00	−	RC113	0.12 ± 0.00	−			
RC053	0.11 ± 0.01	−	RC114	0.20 ± 0.06	−			
RC066	0.97 ± 0.21	−	RC116	0.35 ± 0.09	−			
RC067	0.84 ± 0.31	−	RC117	0.16 ± 0.01	−			
RC068	0.81 ± 0.15	−	RC122	0.20 ± 0.01	−			
RC069	0.15 ± 0.01	−	RC122	0.20 ± 0.01	−			

^a^ Strains were isolated from the crown gall of roses from five regions of S. Korea; ^b^ These means were standardized by the values of reading absorbances of cryptal violet over the optical density of cell growth. std denotes standard deviation; ^c^ The pathogenicity was determined based on the crown gall formation on tomato in the greenhouse of Konkuk University. + denotes for gall formation, – denotes for no gall formation.

## References

[1] Sahin F., Aysan Y. (2003). An Outbreak of Crown Gall Disease on Rose Caused by Agrobacterium Tumefaciens in Turkey. Plant Pathol..

[2] Tzfira T., Citovsky V. (2006). Agrobacterium-Mediated Genetic Transformation of Plants: Biology and Biotechnology. Curr. Opin. Biotechnol..

[3] Christie P.J., Gordon J.E. (2014). The Agrobacterium Ti Plasmids. Microbiol. Spectr..

[4] Pacurar D.I., Thordal-Christensen H., Pacurar M.L., Pamfil D., Botez C., Bellini C. (2011). Agrobacterium Tumefaciens: From Crown Gall Tumors to Genetic Transformation. Physiol. Mol. Plant Pathol..

[5] Guyon P., Chilton M.D., Petit A., Tempe J. (1980). Agropine in "Null-Type" Crown Gall Tumors: Evidence for Generality of the Opine Concept. Proc. Natl. Acad. Sci. USA.

[6] Palanichelvam K., Veluthambi K. (1996). Octopine-And Nopaline-Inducible Proteins in Agrobacterium Tumefaciens are Also Induced by Arginine. Curr. Microbiol..

[7] Dessaux Y., Petit A., Tempe J. (1993). Chemistry and Biochemistry of Opines, Chemical Mediators of Parasitism. Phytochemistry.

[8] Petit A., David C., Dahl G.A., Ellis J.G., Guyon P., Casse-Delbart F., Tempe J. (1983). Further Extension of the Opine Concept: Plasmids in Agrobacterium Rhizogenes Cooperate for Opine Degradation. Mol. Gen. Genet..

[9] Spaink H.P., Kondorosi A., Hooykaas P. (1998). The Rhizobiaceae—Molecular Biology of Model Plant Associated Bacteria.

[10] Kim H.S., Yi H., Myung J., Piper K.R., Farrand S.K. (2008). Opine-Based Agrobacterium Competitiveness: Dual Expression Control of the Agrocinopine Catabolism (ACC) Operon by Agrocinopines and Phosphate Levels. J. Bacteriol..

[11] Palanichelvam K., Oger P., Clough S.J., Cha C., Bent A.F., Farrand S.K. (2000). A Second T-Region of the Soybean-Supervirulent Chrysopine-Type Ti Plasmid pTiChry5, and Construction of a Fully Disarmed Vir Helper Plasmid. Mol. Plant Microbe Interact..

[12] Britton M.T., Escobar M.A., Dandekar A.M. (2008). The Oncogenes of Agrobacterium Tumefaciens and Agrobacterium Rhizogenes. Agrobacterium: From Biology to Biotechnology.

[13] Gohlke J., Deeken R. (2014). Plant Responses to Agrobacterium Tumefaciens and Crown Gall Development. Front. Plant Sci..

[14] Dessaux Y., Faure D. (2018). Quorum Sensing and Quorum Quenching in Agrobacterium: A Go/No Go System. Genes.

[15] Kuzmanovic N., Pulawska J. (2019). Evolutionary Relatedness and Classification of Tumor-Inducing and Opine-Catabolic Plasmids in Three Rhizobium Rhizogenes Strains Isolated from the Same Crown Gall Tumor. Genome Boil. Evol..

[16] Zhu J., Oger P.M., Schrammeijer B., Hooykaas P.J.J., Farrand S.K., Winans S.C. (2000). The Bases of Crown Gall Tumorigenesis. J. Bacteriol..

[17] Habeeb L.F. (1991). Transcription of the Octopine Catabolism Operon of the Agrobacterium Tumor-Inducing Plasmid pTiA6 Is Activated by a LysR-Type Regulatory Protein. Mol. Plant Microbe Interact..

[18] Wang L., Winans S.C. (1995). High Angle and Ligand-Induced Low Angle DNA Bends Incited by OccR Lie in the Same Plane with OccR Bound to the Interior Angle. J. Mol. Boil..

[19] Von Bodman S.B., Hayman G.T., Farrand S.K. (1992). Opine Catabolism and Conjugal Transfer of the Nopaline Ti Plasmid pTiC58 are Coordinately Regulated by a Single Repressor. Proc. Natl. Acad. Sci. USA.

[20] White C.E., Winans S.C. (2007). Cell–Cell Communication in the Plant Pathogen Agrobacterium Tumefaciens. Philos. Trans. R. Soc. B Boil. Sci..

[21] Venturi V., Fuqua C. (2013). Chemical Signaling Between Plants and Plant-Pathogenic Bacteria. Annu. Rev. Phytopathol..

[22] Luo Z.Q., Qin Y., Farrand S.K. (2000). The Antiactivator TraM Interferes with the Autoinducer-Dependent Binding of TraR to DNA by Interacting with the C-Terminal Region of the Quorum-Sensing Activator. J. Boil. Chem..

[23] Haudecoeur E., Faure D. (2010). A Fine Control of Quorum-Sensing Communication in Agrobacterium Tumefaciens. Commun. Integr. Boil..

[24] Chai Y., Zhu J., Winans S.C. (2001). TrlR, a Defective TraR-Like Protein of Agrobacterium Tumefaciens, Blocks TraR Function in Vitro by Forming Inactive TrlR:TraR Dimers. Mol. Microbiol..

[25] Laverde-Gomez J.A., Sarkar M.K., Christie P.J., Vasil M., Darwin A. (2012). Regulation of Bacterial Type IV Secretion Systems. Regulation of Bacterial Virulence.

[26] Morton E.R., Fuqua C. (2012). Phenotypic Analyses of Agrobacterium. Curr. Protoc. Microbiol..

[27] Weisburg W.G., Barns S.M., Pelletier D.A., Lane D.J. (1991). 16S Ribosomal DNA Amplification for Phylogenetic Study. J. Bacteriol..

[28] Hiraishi A. (1992). Direct Automated Sequencing of 16S rDNA Amplified by Polymerase Chain Reaction from Bacterial Cultures Without DNA Purification. Lett. Appl. Microbiol..

[29] Cole J.R., Wang Q., Fish J.A., Chai B., McGarrell D.M., Sun Y., Brown C.T., Porras-Alfaro A., Kuske C.R., Tiedje J.M. (2014). Ribosomal Database Project: Data and Tools for High Throughput rRNA Analysis. Nucl. Acids Res..

[30] Altschul S. (1997). Gapped BLAST and PSI-BLAST: A new generation of protein database search programs. Nucleic Acids Res..

[31] Thompson J.D., Plewniak F., Poch O. (1999). A Comprehensive Comparison of Multiple Sequence Alignment Programs. Nucleic Acids Res..

[32] Saitou N., Nei M. (1987). The neighbor-Joining Method: A New Method for Reconstructing Phylogenetic Trees. Mol. Boil. Evol..

[33] Kumar S., Stecher G., Tamura K. (2016). MEGA7: Molecular Evolutionary Genetics Analysis Version 7.0 for Bigger Datasets. Mol. Boil. Evol..

[34] Adler J., Templeton B. (1967). The Effect of Environmental Conditions on the Motility of Escherichia coli. J. Gen. Microbiol..

[35] Aloni R., Wolf A., Feigenbaum P., Avni A., Klee H.J. (1998). The Never Ripe Mutant Provides Evidence That Tumor-Induced Ethylene Controls the Morphogenesis of Agrobacterium Tumefaciens-Induced Crown Galls on Tomato Stems. Plant Physiol..

[36] Tan B.S., Yabuki J., Matsumoto S., Kageyama K., Fukui H. (2003). PCR Primers for Identification of Opine Types of Agrobacterium Tumefaciens in Japan. J. Gen. Plant Pathol..

[37] Pulawska J. (2010). Crown Gall of Stone Fruits and Nuts, Economic Significance and Diversity of its Causal Agents: Tumorigenic Agrobacterium Spp.. J. Plant Pathol..

[38] Tiwary B.N., Prasad B., Ghosh A., Kumar S., Jain R.K. (2007). Characterization of Two Novel Biovar of Agrobacterium Tumefaciens Isolated from Root Nodules of Vicia faba. Curr. Microbiol..

[39] Pionnat S., Keller H., Hericher D., Bettachini A., Dessaux Y., Nesme X., Poncet C. (1999). Ti Plasmids from Agrobacterium Characterize Rootstock Clones That Initiated a Spread of Crown Gall Disease in Mediterranean Countries. Appl. Environ. Microbiol..

[40] Kalogeraki V.S., Winans S.C. (1995). The Octopine-Type Ti Plasmid pTiA6 of Agrobacterium Tumefaciens Contains a Gene Homologous to the Chromosomal Virulence gene acvB. J. Bacteriol..

[41] Pansegrau W., Schoumacher F., Hohn B., Lanka E. (1993). Site-Specific Cleavage and Joining of Single-Stranded DNA by VirD2 Protein of Agrobacterium Tumefaciens Ti Plasmids: Analogy to Bacterial Conjugation. Proc. Natl. Acad. Sci. USA.

[42] Haas J.H., Moore L.W., Ream W., Manulis S. (1995). Universal PCR Primers for Detection of Phytopathogenic Agrobacterium Strains. Appl. Environ. Microbiol..

[43] Subramoni S., Nathoo N., Klimov E., Yuan Z.C. (2014). Agrobacterium Tumefaciens Responses to Plant-Derived Signaling Molecules. Front. Plant Sci..

[44] Kim H., Farrand S.K. (1998). Opine Catabolic Loci from Agrobacterium Plasmids Confer Chemotaxis to Their Cognate Substrates. Mol. Plant Microbe Interact..

[45] Oger P., Farrand S.K. (2002). Two Opines Control Conjugal Transfer of an Agrobacterium Plasmid by Regulating Expression of Separate Copies of the Quorum-Sensing Activator Gene traR. J. Bacteriol..

[46] Piper K.R., Von Bodman S.B., Hwang I., Farrand S.K. (1999). Hierarchical Gene Regulatory Systems Arising From Fortuitous Gene Associations: Controlling Quorum Sensing by the Opine Regulon in Agrobacterium. Mol. Microbiol..

[47] Oger P., Farrand S.K. (2001). Co-Evolution of the Agrocinopine Opines and the Agrocinopine-Mediated Control of TraR, the Quorum-Sensing Activator of the Ti Plasmid Conjugation System. Mol. Microbiol..

[48] Melnyk C.W. (2017). Connecting the Plant Vasculature to Friend or Foe. New Phytol..

[49] Garfinkel D.J., Nester E.W. (1980). Agrobacterium Tumefaciens Mutants Affected in Crown Gall Tumorigenesis and Octopine Catabolism. J. Bacteriol..

[50] Vicedo B., Penalver R., Asins M.J., Lopez M.M. (1993). Biological Control of Agrobacterium Tumefaciens, Colonization, and pAgK84 Transfer with Agrobacterium Radiobacter K84 and the Tra Mutant Strain K1026. Appl. Environ. Microbiol..

[51] Lang J., Vigouroux A., Planamente S., El Sahili A., Blin P., Aumont-Nicaise M., Dessaux Y., Morera S., Faure D. (2014). Agrobacterium Uses a Unique Ligand-Binding Mode for Trapping Opines and Acquiring A Competitive Advantage in the Niche Construction on Plant Host. PLOS Pathog..

[52] Kado C.I. (2014). Historical Account on Gaining Insights on the Mechanism of Crown Gall Tumorigenesis Induced by Agrobacterium Tumefaciens. Front. Microbiol..

[53] Klapwijk P.M., Scheulderman T., Schilperoort R.A. (1978). Coordinated Regulation of Octopine Degradation and Conjugative Transfer of Ti Plasmids in Agrobacterium Tumefaciens: Evidence for a Common Regulatory Gene and Separate Operons. J. Bacteriol..

[54] Tzfira T., Citovsky V. (2008). Agrobacterium: From Biology to Biotechnolog.

[55] Khan S.R., Su S., Farrand S.K. (2007). Degradation of Acyl-HSLs by AttM Lactonase and its Role in Controlling the Conjugative Transfer of Ti-Plasmids in Agrobacterium Tumefaciens. Plasmid.

[56] Dessaux Y., Guyon P., Petit A., Tempe J., Demarez M., Legrain C., Tate M.E., Farrand S.K. (1988). Opine Utilization by Agrobacterium Spp.: Octopine-Type Ti Plasmids Encode Two Pathways for Mannopinic Acid Degradation. J. Bacteriol..

[57] Schardl C.L., Kado C.I. (1983). Ti Plasmid and Chromosomal Ornithine Catabolism Genes of Agrobacterium Tumefaciens C58. J. Bacteriol..

[58] Nester E. (2014). Beyond My Wildest Expectations. Annu. Rev. Microbiol..

[59] Kim K.S., Baek C.H., Lee J.K., Yang J.M., Farrand S.K. (2001). Intracellular Accumulation of Mannopine, an Opine Produced by Crown Gall Tumors, Transiently Inhibits Growth of Agrobacterium Tumefaciens. Mol. Plant Microbe Interact..

